# The Antidiabetic Drug Ciglitazone Induces High Grade Bladder Cancer Cells Apoptosis through the Up-Regulation of TRAIL

**DOI:** 10.1371/journal.pone.0028354

**Published:** 2011-12-12

**Authors:** Marie-Laure Plissonnier, Sylvie Fauconnet, Hugues Bittard, Isabelle Lascombe

**Affiliations:** 1 Laboratoire de Biologie Cellulaire et Moléculaire, Equipe d'Accueil 3181 – Institut Fédératif de Recherche N°133, Université de Franche – Comté, Faculté des Sciences Médicales et Pharmaceutiques, Besançon, France; 2 CHRU (Centre Hospitalier Régional Universitaire) de Besançon, Service d'Urologie et d'Andrologie, Besançon, France; Hungarian Academy of Sciences, Hungary

## Abstract

**Background:**

Ciglitazone belongs to the thiazolidinediones class of antidiabetic drug family and is a high-affinity ligand for the Peroxisome Proliferator-Activated Receptor γ (PPARγ). Apart from its antidiabetic activity, this molecule shows antineoplastic effectiveness in numerous cancer cell lines.

**Methodology/Principal Findings:**

Using RT4 (derived from a well differentiated grade I papillary tumor) and T24 (derived from an undifferentiated grade III carcinoma) bladder cancer cells, we investigated the potential of ciglitazone to induce apoptotic cell death and characterized the molecular mechanisms involved. In RT4 cells, the drug induced G2/M cell cycle arrest characterized by an overexpression of p53, p21^waf1/CIP1^ and p27^Kip1^ in concomitance with a decrease of cyclin B1. On the contrary, in T24 cells, it triggered apoptosis *via* extrinsic and intrinsic pathways. Cell cycle arrest and induction of apoptosis occurred at high concentrations through PPARγ activation-independent pathways. We show that *in vivo* treatment of nude mice by ciglitazone inhibits high grade bladder cancer xenograft development. We identified a novel mechanism by which ciglitazone kills cancer cells. Ciglitazone up-regulated soluble and membrane-bound TRAIL and let TRAIL-resistant T24 cells to respond to TRAIL through caspase activation, death receptor signalling pathway and Bid cleavage. We provided evidence that TRAIL-induced apoptosis is partially driven by ciglitazone-mediated down-regulation of c-FLIP and survivin protein levels through a proteasome-dependent degradation mechanism.

**Conclusions/Significance:**

Therefore, ciglitazone could be clinically relevant as chemopreventive or therapeutic agent for the treatment of TRAIL-refractory high grade urothelial cancers.

## Introduction

Urothelial carcinoma of the bladder accounts for ∼5% of all cancer deaths in humans. The majority of bladder tumors (75%) are non muscle-invasive at diagnosis and after local surgical therapy, have a high risk of recurrence and a propensity to progress in grade or stage [1]. Muscle invasive tumors (25%) have a poorer prognosis [2] since 50% of patients will relapse with metastatic disease within 2 years of treatment. Despite the advances with surgery and intravesical immuno- and chemotherapy in the management of patients and although new chemotherapeutic agents (tyrosine kinase inhibitors, anti-angiogenic agents…) are used, no improvement in survival has been observed. Thus, developing novel effective chemotherapeutic regimens with fewer side effects are definitely needed to decrease the morbidity and the mortality of urothelial carcinoma.

Thiazolidinediones (TZD), including rosiglitazone, troglitazone, pioglitazone and ciglitazone are a class of insulin-sensitizing drugs. Besides, rosiglitazone and pioglitazone are currently in clinical use for the treatment of type II diabetes for million of patients. These TZD are high-affinity chemically synthesized ligands of Peroxisome Proliferator-Activated Receptor γ (PPARγ) [3], [4]. PPARγ is a ligand-activated transcription factor of the nuclear hormone receptor superfamily. In addition to its known role in regulation of metabolism and inflammation, PPARγ has also been implicated in carcinogenesis based on studies showing its ability to modulate cellular differentiation, proliferation, and apoptosis. Apart from their antidiabetic activity, TZD elicit growth inhibitory effects *in vitro* on cancer cells of diverse tissue origins and *in vivo* [5]. Few studies indicate that pioglitazone or troglitazone exhibited antiproliferative or pro-apoptotic activities against bladder cancer cell lines [6]–[9] and in rat urothelium [10]. Only one study reported that ciglitazone exhibited inhibitory effects on cell number in a series of cell lines modelling metastatic transitional cell carcinoma of the bladder (TSU-Pr1, TSU-Pr1-B1 and TSU-Pr1-B2) [6].

Apoptosis occurs *via* two separate yet interlinked signaling mechanisms: the extrinsic death receptor-induced pathway activated by proapoptotic receptor signals at the cellular surface, and the intrinsic mitochondria-mediated pathway activated by mitochondrial signals from within the cell [11]. The key mediators of apoptosis are caspases, a family of cysteine proteases that cleave a critical set of cellular proteins near specific aspartic acid residues.

Among potential effective anticancer treatments, TRAIL (TNFα-related apoptosis inducing ligand) is a promising anti-neoplastic agent because it induces apoptosis in cancer cells with only negligible effect on normal cells [12]. TRAIL triggers caspase cascade *via* the extrinsic apoptotic pathway through interaction with TRAIL-responsive death receptors (DR), such as DR4 and DR5, leading to the formation of the death-inducing signalling complex (DISC), the recruitment and rapid activation of caspase 8. In type I cells, TRAIL-R-induced caspase-8 activation directly results in downstream caspase activation and apoptosis, whereas in type II cells caspase-8 activation is ineffective and the signal must be amplified through caspase-8-cleavage of the pro-apoptotic Bid protein and the activation of mitochondrial apoptotic pathway [13] through caspase 9 activation. Both caspases 8 and 9 activate the executioner caspase 3, which is the primary activator of apoptotic DNA fragmentation and leads to cancer cell apoptosis [14].

Survivin and cellular FLICE-inhibitory protein (c-FLIP) are downstream and upstream anti-apoptotic regulators of the apoptotic signaling cascade, respectively.

Like other inhibitors of apoptosis (IAP), survivin acts downstream of mitochondria to prevent processing or activation of initiator caspase-9, leading to inhibition of the activity of the effector caspases. However, the role of survivin is not limited to apoptosis inhibition, but also involves the regulation of cell division [15]. C-FLIP is the major protein that prevents caspase 8 from activation by death receptors through binding to Fas-associated death domain and caspase 8 at the DISC. Although multiple splicing isoforms of c-FLIP mRNA have been described, only two of them, c-FLIP_S_ and c-FLIP_L_, have been significantly studied at the protein level. Both proteins can be recruited to the death-inducing signaling complex and inhibit death receptor-mediated apoptosis [16]. Both c-FLIP_L_ and c-FLIP_S_ are quick turnover proteins; thus, their levels are subject to regulation by ubiquitin/proteasome-mediated degradation [17], [18].

Results from preclinical studies with recombinant TRAIL have demonstrated its significant anti-tumor activities, indicating the potential of utilizing TRAIL as an anticancer agent [19]. Despite this proapoptotic role of TRAIL in the transformed cells, many cancer cells develop resistance towards TRAIL-induced apoptosis. Thus, it has been reported that bladder cancer cell lines, such as T24 and HT1376, are TRAIL-resistant [20]. Identification of drugs or agents that can overcome TRAIL resistance and sensitize cancer cells towards TRAIL-induced apoptosis is thus critically important for targeting TRAIL-resistant cancer cells. The combination of TRAIL with radiation and some chemotherapeutic agents [21], [22] have provided limited success, because this combination also resulted in an increase in systemic toxicity.

The present study reported the effects of ciglitazone on RT4 (derived from a well differentiated grade I papillary tumor) and T24 (derived from an undifferentiated grade III carcinoma) bladder cancer cell lines. The choice of these particularly bladder cancer cell lines is relevant since in RT4 cells, P53 is wild type and the mesenchymal marker N-cadherin is not expressed whereas the epithelial marker E-cadherin is detected. Inversely, in T24 cells, P53 is mutated and E-cadherin is absent and replaced by N-cadherin [23]. Thus, depending on differentiated state of the cells, we showed that ciglitazone individual treatment induced G2/M phase cell cycle arrest but triggered apoptosis only in T24 high grade bladder cancer cells through PPARγ activation-independent mechanisms. In addition, for the first time to our knowledge, the treatment of nude mice by ciglitazone significantly impairs the growth of high grade bladder tumor xenografts confirming our *in vitro* results. Most interestingly, the combination of ciglitazone and TRAIL displayed that ciglitazone let TRAIL-refractory T24 cells to respond to TRAIL. For the first time in bladder cancer cells, we revealed that ciglitazone-mediated up-regulation of TRAIL and a marked decrease of c-FLIP and survivin mediated in part through a proteosomal degradation process contribute to ciglitazone-induced cell death and to the sensitizing effect of this TZD on TRAIL-induced apoptosis.

## Results

### Ciglitazone blocks RT4 (low grade) and T24 (high grade) cells in G2/M phase but induces apoptosis only in T24 cells through caspase activation

**Figure 1:**
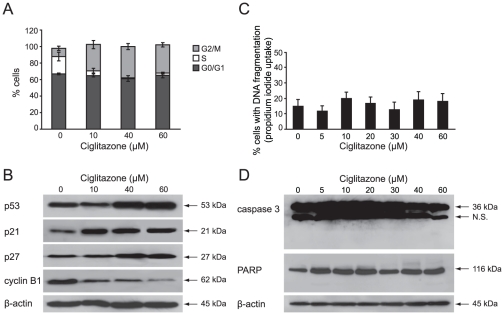
Ciglitazone causes a G2/M cell cycle arrest in RT4 cells. Cells were treated for 24 h with or without ciglitazone at the indicated concentrations. (A) Percentage of cells showing the cell cycle distribution. (C) Hypodiploid DNA content (sub-G1 peak). (B), (D) After treatment, cells were harvested and total protein extracts were subjected to immunoblotting for detection of procaspase 3, PARP, p53, p21^Cip1/Waf1^, p27^Kip1^ and cyclin B1. β-actin was used as an internal loading control. Data shown are representative of 3 independent experiments performed in triplicates.

To investigate the effect of ciglitazone on RT4 and T24 bladder cancer cells apoptosis, cells were treated with varying concentrations of drug (5–60 µM) for 24 h and assessed for propidium iodide stained DNA content using flow cytometric analysis. As shown in Figure 1A, in RT4 cells, the drug induced a dose-dependent increase of cells in the G2/M phase of the cell cycle in association with an increase of expression level of p53, p21^Cip1/Waf1^ and p27^Kip1^ and a decline of cyclin B1 protein level (Figure 1B). But there was no significant increase of the sub-G1 population compared to untreated control cells (Figure 1C) indicating that a 24 h-ciglitazone treatment did not induce apoptosis of these cells even at a high concentration of 60 µM drug. Besides, ciglitazone did not induce cleavage of caspase 3 and its substrate PARP (Figure 1D).

**Figure 2:**
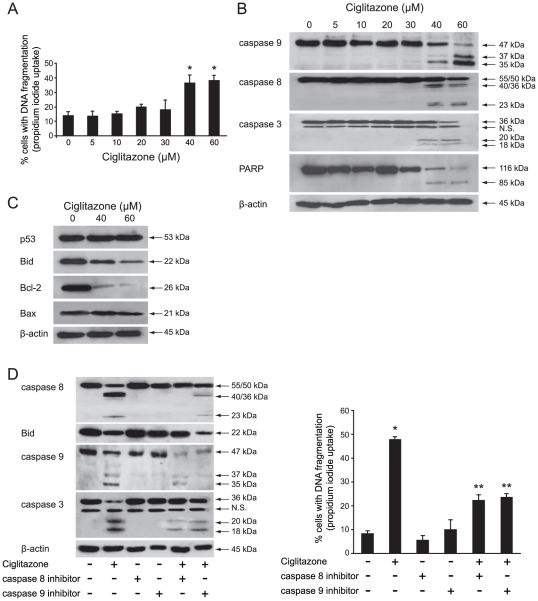
Ciglitazone induces apoptosis in T24 cells. Cells were exposed for 24 h to vehicle or ciglitazone at the indicated concentrations. (A) The percentage of cells showing the hypodiploid DNA content (sub-G1 peak) was evaluated by flow cytometry analysis. (B), (C) The cleavage of caspases 9, 8, 3 and PARP as well as the expression of pro- and anti-apoptotic proteins were determined by western blotting analysis. (D, *left*) Cells were preincubated 1 h with 50 µM caspase 8 (Z-IETD-FMK) or 9 (Z-LEHD-FMK) specific inhibitor before treatment with 40 µM ciglitazone for 12 h and assessment of caspases 8, 9, 3 and Bid processing was analysed by western-blotting. β-actin was used as an internal loading control ; (D, *right*) After indicated treatments, cells were stained with PI for DNA fragmentation analysis by flow cytometry. Data are means ± SD of 3 independent experiments performed in triplicates. *P<0.05, significant differences compared with untreated cells ; **P<0.05, significant differences compared with ciglitazone-treated cells with the use of two-tailed unpaired Student's *t* test.

As observed for RT4 cells, there was a dose-dependent increase of G2/M fraction of T24 cells (data not shown) but unlike RT4 cells, the analysis of nuclear DNA distribution in T24 cells showed a dose-dependent increase of sub-G1 population with ∼40% cell death from 40 µM concentration (Figure 2A). To characterize the cell death pathway triggered by ciglitazone, the cleavage of caspases 9, 8, 3 was determined by western blotting analysis. From a 40 µM concentration of drug, a **considerable** enhanced processing of both caspase 9 and caspase 8 to their active fragments was detected (Figure 2B). Caspase 3 which represents the classical downstream execution enzyme of caspase 8 and 9 was cleaved into its active form (20/18 kDa). Activation of caspase 3 was confirmed by the cleavage of the pro-form PARP (116 kDa) to the inactive form (85 kDa) (Figure 2B). As shown in Figure 2C, while p53 and Bax expression levels were unchanged, Bcl-2 was drastically decreased and Bid was truncated. These results indicated that T24 cells could amplify the apoptotic signal through the mitochondria and behave like type II cells after ciglitazone exposure.

The caspase cascade activation is a key event for apoptotic process. As expected, the incubation with caspase 8 (Z-IETD-FMK) or 9 (Z-LEHD-FMK) specific inhibitors blocked caspases 8 or 9 processing (Figure 2D, left panel) and decreased the ciglitazone-induced apoptosis as evidenced by a significant decrease of sub-G1 population (Figure 2D, right panel) associated with a less caspase 3 cleavage (Figure 2D, left panel). These data indicate that RT4 and T24 cells did not have a similar response to ciglitazone treatment. Indeed, ciglitazone-induced apoptosis was restricted to T24 cells even though it blocked T24 and RT4 cells at the G2/M phase of the cell cycle. In addition, the two main apoptotic pathways, the death receptor and mitochondrial pathways, seem to be activated in T24 cells.

### Ciglitazone did not act through PPARγ transcriptional activation

**Figure 3:**
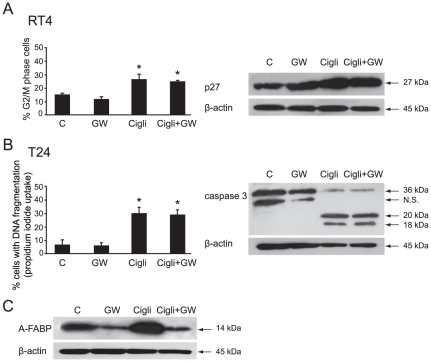
Ciglitazone action is PPARγ activation-independent. RT4 and T24 cells were treated for 24 h by 40 µM of ciglitazone alone or in combination with 80 µM of GW9662, an irreversible potent inhibitor of PPARγ. (A, *left*) Accumulation of RT4 cells in G2/M phase of the cell cycle was analysed by flow cytometry analysis ; (A, *right*) After treatment, whole cell lysates were prepared and 20 µg of total protein extract were subjected to immunoblotting for detection of p27^Kip1^. (B, *left*) The percentage of cells showing hypodiploid DNA content (sub-G1 peak) was evaluated by flow cytometry analysis ; (B, *right*) After treatment, whole cell lysates were prepared and 15 µg of total protein extract were subjected to immunoblotting for detection of procaspase 3. (C) Assessment of the PPARγ antagonist GW9662 efficiency on Adipocyte-Fatty Acid Binding Protein (A-FABP), a known PPARγ target in T24 cells by western-blotting analysis. β-actin was used as an internal loading control. Data are means ± SD of 3 independent experiments performed in triplicates. *P<0.05, significant differences compared with untreated cells with the use of two-tailed unpaired Student's *t* test.

To determine whether PPARγ transactivation was required for ciglitazone-promoted cell cycle arrest or apoptosis, RT4 and T24 cells were treated for 24 h by 40 µM of drug alone or in combination with 80 µM of GW9662, an irreversible potent inhibitor of PPARγ. The action of ciglitazone on G2/M cell cycle arrest in RT4 cells was not inhibited (Figure 3A, left panel). As expected, the increased expression level of p27^Kip1^, observed with ciglitazone, was not affected when GW9662 was associated with the drug (Figure 3A, right panel). The impact of ciglitazone on T24 cell death (Figure 3B, left panel) and caspase 3 cleavage (Figure 3B, right panel) was not reversed in the presence of the PPARγ antagonist. Thus, GW9662 had no inhibitory effect but yet it was effective since in T24 cells, it blocked the overexpression of the A-FABP PPARγ target upon ciglitazone treatment (Figure 3C) as previously reported [24]. Taken together, this set of experiments established that in T24 bladder cancer cells, ciglitazone induced apoptosis through PPARγ activation-independent signalling pathways.

### Ciglitazone up-regulates soluble and membrane-bound TRAIL in T24 cells

**Figure 4:**
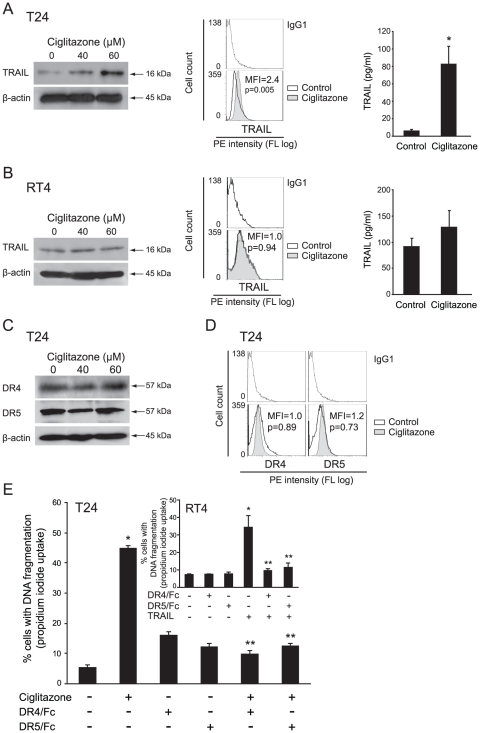
Effect of ciglitazone on soluble and membrane-bound TRAIL and on DR4/DR5 receptors signaling pathway. RT4 and T24 cells were treated for 24 h with ciglitazone at 40 and/or 60 µM. In RT4 (A) and T24 (B) cells, whole cell lysates were assayed for TRAIL expression by western-blotting analysis. β-actin was used as an internal loading control. Cells were stained with anti-TRAIL-PE and analysed by flow cytometry. After 40 µM ciglitazone treatment for 24 h, conditioned media were collected and the concentration of TRAIL was measured by ELISA. Data are means ± SD of 2 independent experiments performed in quadruplicates. *P<0.05, significant differences compared with untreated cells with the use of two-tailed unpaired Student's *t* test. (C) Cellular proteins were isolated from T24 cells and subjected to immunoblotting for detection of DR4 and DR5. β-actin was used as an internal loading control. (D) T24 cells were exposed to 40 µM ciglitazone for 12 h, stained with anti-DR4-PE or anti-DR5-PE and analysed by flow cytometry. (E) T24 cells were preincubated with or without monoclonal antibodies blocking DR4 and DR5 receptors (5 mg/ml) for 1 h and stimulated for 12 h by 40 µM ciglitazone or 50 ng/ml TRAIL for RT4 cells (*insert*). The percentage of cells showing hypodiploid DNA content (sub-G1 peak) was evaluated by flow cytometry analysis. Data are means ± SD of 3 independent experiments performed in triplicates. *P<0.05, significant differences compared with untreated cells ; **P<0.05, significant differences compared with ciglitazone-treated cells with the use of two-tailed unpaired Student's *t* test.

We investigated whether ciglitazone can modulate the expression of TRAIL in T24 and RT4 cells. Upon 40 µM ciglitazone exposure, we observed an increase of TRAIL ligand level in T24 whole cell extracts as assessed by western-blotting, an increase of membrane-bound TRAIL as evidenced by flow cytometry analysis with specific PE-conjugated TRAIL antibody, as well as an increase of TRAIL level in T24 cell conditioned media determined by ELISA (Figure 4A). On the other hand, ciglitazone, which did not induce apoptosis in RT4 cells, did not raise TRAIL in whole cell extracts as well as at the cell surface and in conditioned media (Figure 4B). We thus believe that ciglitzone could cause apoptosis of high grade T24 bladder cancer cells by increasing TRAIL expression.

### Ciglitazone did not affect DR4 and DR5 expression in T24 cells

In T24 cells, we have shown a caspase 8 cleavage and the increase of TRAIL by ciglitazone. To analyse whether ciglitazone-induced cell death is dependent on modification of death receptor expression, cells were treated with 40 or 60 µM drug for 24 h. We demonstrated that ciglitazone did not modify DR4 and DR5 expression level as assessed in whole cell lysates by western-blotting (Figure 4C) as well as their cell surface expression as evidenced by flow cytometry analysis with specific PE-conjugated antibodies (Figure 4D). To investigate whether ciglitazone-triggered apoptosis was a function of death receptor signalling amplification, DR4 and DR5 stimulation was blocked by pre-incubation of cells with blocking anti-DR4 or anti-DR5 antibodies before drug treatment. The inactivation of both DR4 and DR5 significantly decreased the ciglitazone-promoted apoptosis (Figure 4E). To validate the death receptor blocking antibodies efficiency at the concentrations used, we exposed TRAIL-sensitive RT4 cells to TRAIL as a positive control (Figure 4E, insert). As expected, inactivating each of the receptors completely inhibited TRAIL-induced apoptosis. These data display that ciglitazone activates the extrinsic apoptotic pathway through DR4 and DR5.

### Ciglitazone inhibits bladder tumor xenograft development in nude mice

**Figure 5:**
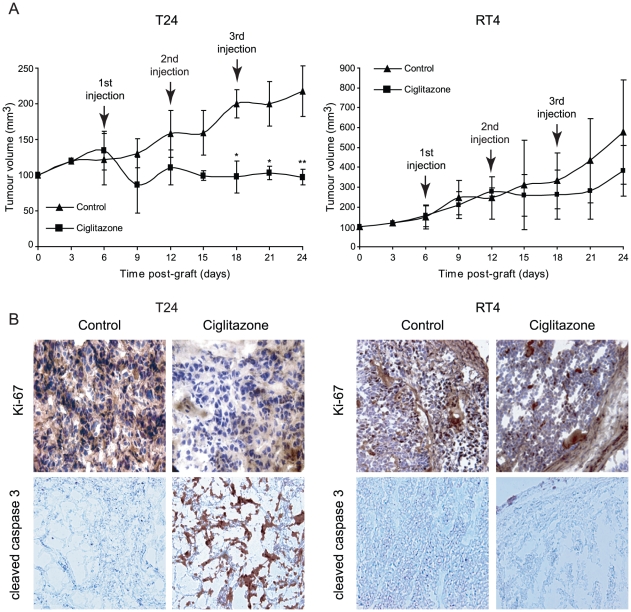
Antitumor activity of ciglitazone administered as monotherapy in athymic mice bearing T24 and RT4 bladder cancer xenografts. Mice were inoculated subcutaneously with exponentially growing T24 and RT4 bladder cancer cells (5×10^6^ cells). When tumor size reached 40 mm^3^ in volume, mice were randomly divided into control and treated groups (n = 10). Intraperitoneal injections of ciglitazone were weekly administered at a dose of 15 mg/kg during three weeks. Control animals received only saline vehicle following an identical schedule. (A) The growth tumor curves were determined by measuring the tumor volume. *P<0.05, significant differences compared with untreated animals with the use of two-way ANOVA test (evaluation of the tumor volume development over time) ; **P<0.05, significant differences between control and treated mice at each post-graft time with the use of two-tailed unpaired Student's *t* test. (B) Immunohistochemical staining of representative paraffin-embedded sections of tumors from untreated or ciglitazone-treated mice. Sections were fixed and stained for Ki-67 and active caspase 3. Each panel is representative of 5 sections for each of ten tumors from control and ciglitazone-treated mice. Original magnification, ×20.

To investigate the antitumor effect of ciglitazone *in vivo* (Figure 5), T24 and RT4 cells as well were subcutaneously injected in 6-week-old-female athymic nude mice. When palpable tumors were formed, mice were treated by vehicle or ciglitazone (15 mg/kg) at the rate of one injection per week for three weeks. Tumor volume was measured twice per week until the mice were sacrificed. The tumor incidence was 100% in all animals. The volume of the tumors in T24 cells-grafted mice did not increase in ciglitazone-treated animals compared to vehicle-treated control animals. There was a significant difference from the third injection of ciglitazone (*P*<0.05) (Figure 5A, left panel). In RT4 cells-grafted mice, tumor volume seemed to develop less than in control mice upon ciglitazone treatment but this was not statistically significant (Figure 5A, right panel). To determine whether the inhibition of the tumor development by ciglitazone is due to inhibition of proliferation, apoptosis, or both, we evaluated Ki67 expression and caspase 3 activation on tumor tissue sections by immunohistochemical analysis. The number of Ki67-positive cells was lower in T24 and RT4 cells-xenografted tumor sections from ciglitazone-treated animals compared to tumor sections from untreated animals. On the contrary, there was no detectable activated caspase 3 in RT4 cells-xenografted tumor sections and a strong activated caspase 3 staining in T24 cells-xenografted tumor sections from ciglitazone treated animals compared to tumor sections from untreated animals (Figure 5B). These results indicate, for the first time to our knowledge, that the significant reduction of tumor volume observed in ciglitazone-treated mice (xenografted with T24 bladder cancer cells) requires the inhibition of proliferation as well as the induction of apoptosis.

### Ciglitazone and TRAIL combined treatment induces synergistic apoptosis through death receptor signalling pathway, caspase activation and Bid cleavage

**Figure 6:**
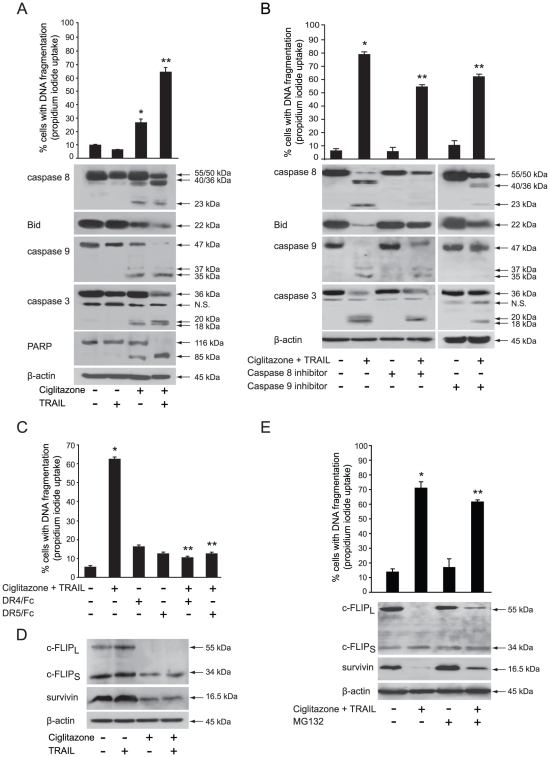
Ciglitazone lets TRAIL-resistant T24 cells to respond to TRAIL through caspases activation and c-FLIP and survivin downregulation. T24 cells were treated or not with 40 µM ciglitazone for 24 h or human recombinant TRAIL (50 ng/ml) for 12 h or cotreated with ciglitazone and TRAIL (added here for the last 12 h). (A, *top*) The percentage of cells showing hypodiploid DNA content (sub-G1 peak) was evaluated by flow cytometry analysis. Data are means ± SD of 3 independent experiments performed in triplicates. *P<0.05, significant differences compared with untreated cells; **P<0.05, significant differences compared with individual treatment-exposed cells with the use of two-tailed unpaired Student's *t* test; (A, *bottom*) Immunoblotting detection of caspases 8, 9 and 3, PARP and Bid. (B) Cells were preincubated 1 h with 50 µM caspase 8 (Z-IETD-FMK) or 9 (Z-LEHD-FMK) specific inhibitor before indicated treatment; *top*, the percentage of cells showing hypodiploid DNA content was evaluated by flow cytometry analysis; *bottom*, caspases 8, 9, 3 and Bid cleavage was assayed by western-blotting analysis. (C) Cells were preincubated with or without monoclonal antibodies blocking DR4 and DR5 receptors for 1 h and stimulated as indicated. The percentage of cells showing hypodiploid DNA content was evaluated by flow cytometry analysis. (D) Immunoblotting detection of c-FLIP and survivin from T24 whole cell lysates. (E) Cells were pretreated with 25 µM proteasome inhibitor MG132 for 30 min and stimulated with 40 µM ciglitazone for 12 h ; *top*, the percentage of cells showing hypodiploid DNA content was evaluated by flow cytometry analysis ; *bottom*, immunoblotting detection of c-FLIP and survivin. β-actin was used as an internal loading control. (B, C, E) Data are means ± SD of 2 independent experiments performed in triplicates. *P<0.05, significant differences compared with untreated cells ; **, *P*<0.05, significant differences compared with ciglitazone and TRAIL cotreated cells with the use of two-tailed unpaired Student's *t* test.

T24 cells are known to be highly resistant to TRAIL after exposure to increasing concentrations of exogenous soluble recombinant human TRAIL ranging from 10 to 200 ng/ml [25]. According to our results demonstrating that ciglitazone up-regulates TRAIL expression, we postulated that ciglitazone kills T24 cells by restoring their sensitivity to TRAIL-induced apoptosis. To validate this hypothesis, cells were treated with vehicle or 40 µM ciglitazone for 24 h or 50 ng/ml recombinant TRAIL for the last 12 h alone or in combination. As previously reported, we showed also that T24 cells were refractory to TRAIL-induced apoptosis. More interestingly, there was a significant increase of cell death in ciglitazone and TRAIL-cotreated cells compared to ciglitazone alone-treated cells (Figure 6A, top). The TRAIL-sensitizing action of ciglitazone resulted in pro-caspase 8 and 3 processing and PARP proteolysis but involved also the mitochondria amplification loop as indicated by caspase 9 and Bid cleavage (Figure 6A, bottom). In the presence of caspase 8 (Z-IETD-FMK) or caspase 9 (Z-LEHD-FMK) specific inhibitors, we observed an inhibition of either caspase 8 or caspase 9 processing and the absence of Bid cleavage as well, in ciglitazone and TRAIL cotreated cells (Figure 6B, bottom). This was associated with a decrease of cell death in the presence of caspase inhibitors as evidenced by a significant decrease of DNA fragmentation (Figure 6B, top). To confirm the involvement of death receptor activation, DR4 and DR5 stimulation was inhibited by pre-incubation of cells with blocking anti-DR4 or anti-DR5 antibodies before treatment (Figure 6C). Inactivating each of the receptors upon TRAIL and ciglitazone cotreatment efficiently blocked cell death confirming that apoptosis was mediated through specific interactions between TRAIL and its receptors.

### Ciglitazone down-regulates c-FLIP and survivin through a proteasome-dependent degradation

We further investigated the effect of ciglitazone and the association of ciglitazone with TRAIL on the expression of apoptosis inhibitors such as survivin and c-FLIP known to be involved in the regulation of both intrinsic and extrinsic apoptotic pathways. As presented in Figure 6D, ciglitazone, as individual treatment or in combination with TRAIL, reduced both c-FLIP_S_ and survivin protein levels to the same extent and dramatically down-regulated c-FLIP_L_. On the contrary, TRAIL single treatment did not affect **considerably** c-FLIP and survivin protein levels. These findings indicate that down-regulation of c-FLIP and survivin likely contributes to ciglitazone-triggered apoptosis and to ciglitazone-mediated sensitization to TRAIL-induced apoptosis.

The ubiquitine-proteasome pathway plays a central role in the regulation of cell cycle control and apoptosis [26]. Several anti-apoptotic proteins such as c-FLIP and survivin represent prominent targets of the proteasome. To determine whether the ciglitazone-induced down-regulation of such apoptosis negative regulators is mediated through this process, we investigated the effects of the proteasome inhibitor MG132. MG132 restored the levels of survivin and c-FLIP (Figure 6E, bottom) indicating that down-regulation of both apoptosis inhibitors was proteasome dependent. As shown in the Figure 6E (top) the sub-G1 cell population level in cotreated cells was significantly decreased in the presence of MG132. Thus, the recovery of c-FLIP and survivin, that significantly attenuated ciglitazone and TRAIL combination-induced apoptosis, was required but not sufficient to overcome cotreatment-promoted apoptosis in T24 cells.

## Discussion

The present report demonstrated that ciglitazone induced cell growth inhibition through cell cycle course control or induction of apoptosis according to the tumor grade of bladder cancer-derived urothelial cells. Apoptotic effects occurred at high concentrations (40–60 µM) independently of PPARγ activation as already reported in other cellular models [27]. Several potential PPARγ-activation independent mechanisms that may induce apoptosis have been proposed. They include the generation of reactive oxygen species [28], the induction of cellular acidosis [29], the involvement of early growth response-1 and NSAID-activated-gene 1 in colon cancer [30].

For the first time in malignant urothelial cells, we present evidence that ciglitazone induced G2/M cell cycle arrest and changes in cell cycle regulators. The major regulator of the G2 to M transition is the MPF (M-phase promoting factor), which comprises the catalytic subunit Cdc2 and the regulatory subunit cyclin B1. Several observations suggest that p21^Cip1/Waf1^ plays an important role in G2/M checkpoint control. P21^Cip1/Waf1^ inhibits either Cdc2 kinase activity or prevent its activation by directly binding to Cdc2, thereby blocking the interaction of cyclin B1-Cdc2 complexes with their substrates [31]. Our results show that in low grade papillary bladder tumor-derived RT4 cells, ciglitazone led to the down-regulation of cyclin B1 protein level and a marked increase in p21^Cip1/Waf1^ and p27^Kip1^ expression associated with a raise of p53 protein level. P21 is normally induced by p53. P53 has been identified as a direct target of PPARγ [32] and is a potent inhibitor of cellular proliferation. Its expression leads to G1 phase arrest only [33] but some studies reported that p53 hinders G2/M progression in the rat cell line REF52 [34], in a human ovarian cancer cell line [35] and in skin cells [36]. A recent study demonstrated that a prolonged exposure of ciglitazone (72 h) increased p27^Kip1^ protein level through both induction of p27^Kip1^ gene transcription as well as inhibition of p27^Kip1^ protein proteasome-mediated degradation [37]. No PPRE or similar elements have been identified in the promoter. Besides, in RT4 cells, p27^Kip1^ up-regulation was not abolished by PPARγ specific antagonist arguing for the involvement of a PPARγ activation-independent mechanism. Ciglitazone could regulate p27^Kip1^ gene transcription through binding of Sp1 transcription factor on Sp1 binding elements located in the promoter region as described for 1,25 vitamin D3 in U937 cells [38].

Ciglitazone induced G2/M cell cycle arrest in both RT4 and T24 cells but triggered apoptotic cell death only in T24 cells through a PPARγ-activation independent pathway. Very few studies are available on the effect of ciglitazone in bladder cancer cells. Chaffer *et al.* (2006) showed that this antidiabetic drug caused a small decrease in cell number of TSU-Pr1/TSU-Pr1-B1/TSU-Pr1-B2 cell lines after 24 h exposure, which subsided to a modest 10–15% inhibition over days 3 to 9. [39] reported that ciglitazone induced cell death in HT-1376 and 5637 bladder cancer cell lines. Here, we deepened and revealed the underlying mechanisms involved in ciglitazone-induced apoptosis in high grade T24 bladder cancer cells. Our data support the involvement of both intrinsic and extrinsic apoptotic signalling pathways as evidenced by cleavage of caspases 9, 8 and 3 as well as the caspase substrate PARP and the caspase processing blockage with the caspase 8 and 9 specific inhibitors. The cleavage of Bid protein in T24 cells suggests that processing/activation of procaspase 8 drives the caspase cascade with the requirement of the loop amplification from the mitochondria. Most importantly, our study provides first *in vitro* evidence of a novel mechanism by which ciglitazone induces apoptosis through soluble and membrane-bound TRAIL expression up-regulation leading to activation of DR4 and DR5 since their knockdown by specific blocking antibodies significantly decreased ciglitazone-triggered apoptosis demonstrating their critical role in this process. Only one study, carried out in animal cells and more particularly in rat GH-secreting (GH3) cells, revealed that ciglitazone-induced apoptosis was associated with an increased expression of TRAIL assessed by western-blotting analysis in whole cell extracts [40].

Malignant urothelial cells showed differential apoptotic susceptibilities or resistance to TRAIL-induced apoptosis. We showed that T24 cells were resistant to TRAIL as already reported [20], [25]. Oka *et al.* (2006) revealed that this protection from TRAIL-induced apoptosis was related to a high expression of constitutively active Akt. Here, we demonstrated that ciglitazone sensitized cells to exogenous TRAIL. This result could have an important impact for bladder cancer treatment. Indeed, TRAIL could play a role in BCG-induced antitumor effects [41]. BCG endovesical instillations are effective against bladder tumor recurrence. According to some randomized controlled trials, only BCG can curb progression. It has been postulated that BCG stimulates the immune system by activating macrophages, T lymphocytes, B lymphocytes, thus augmenting the host response to tumor surveillance [42]. Functional TRAIL has been detected in the urine of patients undergoing BCG therapy and increased urine levels in those patients who responded to BCG immunotherapy compared with non responders [41]. Thus, ciglitazone could improve and potentiate BCG therapy in responders and sensitize to BCG therapy in non responders.

The underlying mechanisms explaining how thiazolidinediones sensitize cells to TRAIL-induced cell death remain poorly understood but could involve the down-regulation of inhibitors of apoptosis proteins [43], the ROS-mediated up-regulation of DR5 [36] or the caspases 3 and 8-mediated β-catenin cleavage [44]. It has been documented that some TZD decrease c-FLIP [17] or survivin expression [45]. Interestingly, the present findings propose that a down-regulation of c-FLIP was associated with apoptosis induced by ciglitazone combined with TRAIL and the reduction in survivin level could be partly responsible for the action of ciglitazone on the TRAIL-promoted apoptosis in T24 cells. Survivin is expressed at high levels in many tumors and has been described as a prognostic marker for bladder cancer progression [46]. Survivin inhibits mitochondrial apoptosis but is able to block TRAIL-induced cell death. This can be explained by cross-links between extrinsic and intrinsic death pathways through the Bid protein cleavage as detected upon ciglitazone treatment in T24 cells. Survivin is a bifunctional protein. In addition to act as an apoptosis inhibitor, survivin is a key regulator of mitosis and is involved in G2/M transition. Besides, siRNA mediated down-regulation of survivin in bladder cancer cell lines, such as RT4 and T24, was associated with a specific G2/M arrest [47], [48]. Now, we showed that ciglitazone increased the fraction of RT4 and T24 (data not shown) cells in G2/M phase of the cell cycle. CDK1/cyclin B1 is responsible for regulating survivin through phosphorylation of Thr34 [49]. Thus, we could hypothesize that ciglitazone led to the survivin down-regulation by acting on CDK1/cyclin B1 complex. The decrease of cyclin B1 associated with the RT4 and T24 cells accumulation in G2/M phase of the cell cycle could prevent the phosphorylation and accumulation of survivin, removing a viability checkpoint through which it leads to apoptosis in the case of T24 cells. Further experiments are needed to clarify this point.

Several mechanisms explain TZD-mediated down-regulation of downstream (survivin) and upstream (c-FLIP) anti-apoptotic regulators of the apoptotic signalling cascade namely, ubiquitination and proteasome-dependent degradation with or without transcriptional events [43], [50]. Here, despite the recovery of survivin and c-FLIP protein level after MG132 pretreatment, T24 cells did not completely rescue from cell death. The down-regulation of both proteins is mediated partially through proteasomal degradation because in the presence of MG132, the protein levels did not reach those of untreated cells and this was not sufficient to prevent ciglitazone-induced apoptosis. Further investigations are needed to better understand the molecular mechanisms involved in this process.

We have evaluated the potential anti-urothelial cancer activity of ciglitazone in a mouse model of bladder cancer xenografts. Importantly, our results showed that ciglitazone significantly reduced the development of bladder tumors in mice xenografted with high grade T24 cells but not in mice xenografted with low grade RT4 cells. In addition, ciglitazone did not appear to induce toxicity since the body weight and overall appearance of mice given the drug were not different from those of controls (data not shown). These data demonstrate that ciglitazone has a strong anti-urothelial cancer activity *in vivo*. The immunohistochemical analysis of caspase 3 activation suggests that *in vivo* ciglitazone effects are cytotoxic and correlated with the induction of apoptosis. So, to observe a significant reduction of bladder tumor development, both inhibition of proliferation and induction of apoptosis are needed.

Collectively, this work has identified a novel pathway for the antitumor properties of ciglitazone in human bladder cancer cells. We demonstrate for the first time that ciglitazone is able to reduce bladder cancer development in an *in vivo* model. According to our study, it is needed to elucidate in bladder cancer the distinct PPARγ-independent mechanisms of ciglitazone action prior to clinical exploration. Furthermore, animal studies are warranted in combination of ciglitazone and TRAIL to strengthen our *in vitro* results.

We suggest that endovesical instillations of ciglitazone could be used as individual treatment or in combination with TRAIL or BCG therapy. This could be a potential efficient treatment tool for bladder cancer.

## Materials and Methods

### Reagents

The PPARγ agonist Ciglitazone and the PPARγ antagonist GW9662 were provided from Cayman Chemical. Soluble recombinant TRAIL, proteasome inhibitor MG132, caspase 9 (Z-LEHD-FMK) specific inhibitor and propidium iodide (PI) were purchased from Sigma. Caspase 8 (Z-IETD-FMK) inhibitor was from Bachem (Heidelberg, Germany). The final concentration of organic solvent (dimethylsulfoxide) was less than 0.1% which had no effect on cell viability. DR4 and DR5 blocking antibodies were from Alexis Biochemicals (Lausanne, Switzerland).

### Cell lines, culture conditions, and treatment

The human bladder tumor cell lines RT4 and T24 were obtained from ATCC. Cells were maintained in Mc COY's 5a medium supplemented with 10% fetal calf serum (FCS) (Invitrogen, Cergy Pontoise, France), 1% antibiotic antimycotic mixture (10 mg/ml streptomycin, 10 000 units/ml penicillin, 25 µg/ml amphotericin B), 2 mM glutamine and 15 mM Hepes (Sigma) at 37°C in a humidified 5% CO_2_, 95% O_2_ air incubator. Cells were tested for the absence of mycoplasma before the beginning of experiments. RT4 cells (4.2×10^4^ cells/cm^2^) and T24 cells (5.2×10^3^ cells/cm^2^) were plated in 12-well plates for flow cytometry analysis and in 6-well plates for protein extraction and cultured in 5% FCS culture medium. After 24 h attachment, cells were washed with serum-free culture medium and then treated either with vehicle or with ciglitazone (5 to 60 µM) for 24 h or with TRAIL (20, 50, 100 ng/ml) for 12 h, alone or in combination for 24 h in a serum-free culture medium. In that case, TRAIL was added the last 12 h in the culture medium.

### DNA fragmentation and cell cycle analyses

The distribution of cells in the sub-G1, G0/G1, S and G2/M cell cycle phases was determined by flow cytometry. After ciglitazone treatment, RT4 and T24 cells were detached with trypsin/EDTA, washed in phosphate-buffered saline (PBS) and centrifuged at 1 200 rpm for 10 min. Total population of cells, including floating and adherent cells, was fixed overnight at 4°C by cold absolute ethanol. After fixation followed by washes with PBS/5 mM EDTA, cells were stained with 1 mg/ml propidium iodide in 50 µl PBS/5 mM EDTA complemented with 1 mg/ml RNAse A DNAse-free (Sigma) and incubated at room temperature for 15 min. The DNA content was analysed by flow cytometry using an excitation wavelength set at 488 nm and emission at 610 nm (FC 500, Beckman Coulter). Twenty thousand events were analysed per sample and apoptosis was determined from the Sub-G1 DNA content with CXP software (Beckman Coulter). The percentages of nuclei in the G0/G1, S, G2/M phases of the cell cycle were quantified using Multicycle analysis software (Beckman Coulter). Gating was set to exclude cell debris, doublets, and clumps.

### Western blotting

After ciglitazone treatment, cells were washed with cold PBS and scraped in 120 µl of lysis buffer RIPA (50 mM Tris-HCl pH 7.4, 150 mM NaCl, 1 mM EDTA, 1% Nonidet P40, 0.5% sodium desoxycholate) supplemented with protease inhibitors (Roche, Meylan, France). Then, cell lysates were sonicated and centrifuged at 10 000 rpm for 10 min at 4°C. Protein concentration was estimated using the Bio-Rad protein assay (Bio-Rad, Marnes-la-Coquette, France). Protein extracts (20 µg for RT4 cells or 15 µg for T24 cells) were solved in Laemmli buffer (Bio-Rad) and separated by a 7.5–15% SDS-PAGE. Proteins were transferred onto PVDF membranes (GE Healthcare, Buckinghamshire, UK) and non specific binding was blocked in TBS-Tween 20 buffer (0.5 mM Tris-HCl, 45 mM NaCl, 0.05% Tween 20, pH 7.4) containing 5% nonfat milk. Membranes were incubated for 1 h with the following appropriate primary antibodies: mouse monoclonal anti-caspase 8 (clone 3-1-9, 1∶1 000), anti-p21^Cip1/Waf1^ (6B6, 1∶500), anti-p27^kip1^ (G173-524, 1∶500), anti-p53 (DO-1, 1∶500), anti-cyclin D1 (G124-326, 1∶1 000) and anti-PARP (4C10-5, 1∶1 000) were obtained from BD Pharmingen. Anti-mouse A-FABP (#AF1443, 1∶1 000) was from R&D Systems (Abingdon, UK). Mouse monoclonal cyclin B1 (1∶1 000) was kindly gifted by Dr. Marchini (DKFZ, Heidelberg). Rabbit polyclonal anti-caspase 9 (1∶1 000), mouse monoclonal anti-survivin (1∶500) and mouse monoclonal Bid (1∶500) were from Cell Signaling. Rabbit polyclonal anti-caspase 3 (1∶1 000) was from Stressgen (Victoria, Canada) and mouse monoclonal anti-c-FLIP (NF6, 1∶500) was from Alexis Biochemicals. Rabbit polyclonal anti-DR4 (1∶1 000), anti-DR5 (1∶1 000) and anti-TRAIL (1∶500) were purchased from Chemicon International Inc. Protein blots were probed with mouse monoclonal anti-β-actin (AC-15, Sigma) as controls for protein loading. Bound primary antibodies were detected using HRP-conjugated secondary antibodies: anti-rabbit IgG (1∶10 000) or anti-mouse IgG (1∶10 000) provided from BD Pharmingen. Proteins were visualized by using enhanced chemiluminescence detection method (GE Healthcare) followed by film exposure (Hyperfilm ECL, GE Healthcare).

### Assessment of cell surface DR4, DR5 and TRAIL expression

Cells were treated with ciglitazone alone or in combination with TRAIL as described above. They were detached with 5 mM EDTA and centrifuged at 1 200 rpm for 10 min. Cells (1.10^6^) were resuspended in 50 µL of PBS and stained with 10 µL of phycoerythrin (PE)-conjugated mouse monoclonal anti-human DR4 (clone B-N36), DR5 (clone B-K29) or TRAIL (clone B-S23) provided by Diaclone (Besançon, France), for 30 min at 4°C in the dark. Cells were then washed twice in 1% BSA-PBS and resuspended in 50 µL of PBS. Flow cytometry analyses were performed at a 580 nm wavelength. PE-conjugated mouse IgG1 was used as an isotype control.

### Determination of TRAIL production

RT4 and T24 cells were seeded in triplicates in 6-well plates and cultured in Mc COY's 5a medium with 5% FCS for 24 h. The following day, the cells were stimulated in serum-free medium with 40 µM ciglitazone for 24 h. Supernatants were collected and TRAIL was quantified by enzyme-linked-immunosorbent assay (Diaclone).

### Tumor xenograft model

Five to eight-week-old female *nu/nu* nude mice were maintained according to European Union guidelines for use of laboratory animals. *In vivo* experiments were performed in compliance with the French guidelines for experimental animal studies. Experimental protocols were approved by the Departmental Direction of the Veterinary Division of the Doubs, France (authorization certificate N°25-27/Approval ID: B 25-056-7). Exponentially growing human bladder cancer cell lines RT4 and T24 were subcutaneously injected into the right flank of female nude mice as follows: 5×10^6^ cells in 50 µL PBS with 25% decomplemented FCS for both cell lines but with 50 µL Matrigel (BD Biosciences) for T24 cells. Animals were examined twice a week for tumor development. When tumor size reached 40 mm^3^ in volume, mice were randomly divided into control and treated groups containing ten mice each. Intraperitoneal injections of ciglitazone were weekly administered at a dose of 15 mg/kg during three weeks. Control animals received only saline vehicle following an identical schedule. Tumor size was monitored twice a week by measuring length and width with a calliper. Tumor volume was estimated using the following formula: volume = width^2^×length×½. Mice were sacrificed one week after the third injection and tumors were removed.

### Immunohistochemistry

Vehicle- and ciglitazone-treated tumors were collected, fixed in 4% formalin and paraffin-embedded. Then, blocks were cut serially at 5 µm thick, deparaffinized in toluene, rehydrated in graded ethanol. Antigen retrieval was achieved by micro-wave treatment in 0.5 M Tris-buffered saline (pH 6.0) at 750 W for 30 minutes. Sections were then incubated with primary antibodies at room temperature using an automated immunohistochemical processor (Techmate 500 Plus, DakoCytomation, Trappes, France) according to the manufacturer's instructions. The biotinylated primary antibody anti-Ki-67 (DakoCytomation, MIB-1, 1∶150) was used for evaluation of proliferating cells. Activated caspase 3 was detected with Apoptosis marker: SignalStain cleaved Caspase 3 (Asp175) IHC Detection kit (Cell Signaling). Slides were then treated with alkaline phosphatase-conjugated avidin. Finally, alkaline phosphatase activity was revealed by NBT-BCIP. Sections were counterstained with Harris' hematoxylin, dehydrated through alcohol and mounted using a standard procedure. Negative controls were obtained by omitting the first antibody. Slides were then examined and pictures were taken using Zeiss Axioskop 40 photomicroscope.

### Statistical analyses

For *in vitro* experiments, two-tailed unpaired Student's *t* test was used to examine the significant differences between groups. Data are expressed as mean ± SD of three independent experiments or as specified for each figure. The *in vivo* therapeutic efficacy of ciglitazone was assessed by evaluating the tumor volume development over time with two-way ANOVA test. Differences between control and treated mice at each post-graft time were determined by Student's *t* test. Values with *P*<0.05 were considered significant.
